# TRAPPC2l Participates in Male Germ Cell Development by Regulating Cell Division

**DOI:** 10.1111/cpr.13810

**Published:** 2025-01-26

**Authors:** Mengyue Wang, Jiayi Li, Bowen Liu, Zhiming Shen, Min Chen, Xiuhong Cui, Hongbin Liu, Fei Gao, Han Zhao

**Affiliations:** ^1^ State Key Laboratory of Reproductive Medicine and Offspring Health, Center for Reproductive Medicine, Institute of Women, Children and Reproductive Health Shandong University Jinan China; ^2^ National Research Center for Assisted Reproductive Technology and Reproductive Genetics Shandong University Jinan China; ^3^ State Key Laboratory of Stem Cell and Reproductive Biology, Institute of Zoology Chinese Academy of Sciences Beijing China; ^4^ Institute for Stem Cell and Regeneration Chinese Academy of Sciences Beijing China; ^5^ Guangdong Cardiovascular Institute, Guangdong Provincial People's Hospital Guangdong Academy of Medical Sciences Guangzhou China; ^6^ Beijing Institute for Stem Cell and Regenerative Medicine Beijing China

**Keywords:** Gonocyte, meiosis, non‐obstructive azoospermia (NOA), *Trappc2l*

## Abstract

TRAPPC2L is a core subunit of the Transport Protein Particle (TRAPP) complex, which is involved in vesicle transport and autophagy. Mutations in *Trappc2l* gene are associated with neurodevelopmental disorders, characterised by severe neurodevelopmental delays and varying degrees of muscle abnormalities. In this study, we found that the knockout of *Trappc2l* did not cause developmental abnormalities in both male and female mice. However, the male mice were completely infertile. Histological examination revealed that germ cell syncytial structures with multiple nuclear were formed in *Trappc2l* knockout mice from embryonic day 17.5 (E17.5) and the number and size of these structures gradually were increased at later developmental stages. The germ cells were completely lost at 2 weeks after birth. Further study found that germ cell syncytial structures were most likely formed by abnormal cell division but not cell fusion. We also found that meiosis‐associated genes *Stra8* and *Sycp3* were expressed in *Trappc2l‐*deficient germ cells during the embryonic stage. Our study demonstrated that *Trappc2l* is essential for germ cell development in male mice which is probably involved in keeping the mitotic quiescent state of male germ cells during the embryonic stage.

## Graphical abstract

*Trappc2l* is crucial for maintaining the quiescent state of male gonocytes. In the absence of *Trappc2l*, the quiescent state of gonocytes is disrupted, leading to premature and abnormal initiation of meiosis, which consequently results in subsequent abnormalities in sperm development.

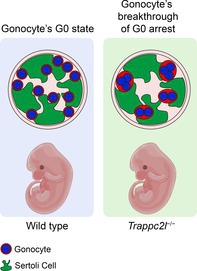

## 1 Introduction

Infertility is a multifactorial pathological condition affecting approximately 10%–15% of couples worldwide, and nearly one‐half of infertility cases involve male infertility [1, 2]. Non‐obstructive azoospermia (NOA) is the most severe form of male infertility, comprising about 60% of azoospermia cases, with approximately 27% having a genetic basis [3]. Studying germ cell development is essential for understanding male infertility and has significant implications for clinical diagnosis and treatment [4]. The germ cells are derived from primordial germ cells (PGCs) which are formed during the early stage of embryonic development. PGCs colonise into the genital ridge at approximately E10.5 [5, 6, 7]. After sex determination, the germ cells in male and female gonads undergo different developmental pathways. Female germ cells initiate the process of meiosis right and arrest at the diplotene stage [8]. By contrast, male germ cells undergo both mitotic and meiotic arrest during the embryonic stage and start meiosis and mitosis after birth. However, the underlying mechanisms which regulate the mitotic arrest of male germ cells during embryonic remain unkonwn [9, 10, 11, 12].

Transport Protein Particle (TRAPP) is a multi‐subunit protein complex which is involved in vesicle transport during intracellular secretion and autophagy [13]. TRAPP complexes are generally categorised into three types: TRAPP I, TRAPP II and TRAPP III [14, 15, 16, 17]. TRAPP I primarily regulates vesicle transport from the endoplasmic reticulum (ER) to the Golgi apparatus. TRAPP III is formed by adding the TRAPP III‐specific subunit Trs85 to TRAPP I, facilitating autophagy and vesicle transport from the ER to the Golgi [18, 19]. TRAPP II is formed by adding the TRAPP II‐specific subunits Trs130, Trs120 and Trs65 to TRAPP I, regulates vesicle transport within the Golgi and from the Golgi to the plasma membrane and participates in autophagy [20]. TRAPPC2L is a core subunit of the TRAPP II and III complexes, playing a critical role as a tethering factor in membrane transport [21].

Previous research has found that TRAPPC2L functions as a bridging protein and facilitates the assembly of the TRAPP complex through interacting with the core TRAPP protein TRAPPC6a^22^. TRAPPC2L also interacts with TRAPPC10/Trs130, activating GTPase RAB11 [20], which binds to downstream target proteins and promotes vesicle transport, thereby controlling intracellular vesicle trafficking through a molecular switch mechanism. Mutations in TRAPPC2L disrupt RAB11 activity, impairing membrane transport and maintenance, ultimately leading to neurodevelopmental disorders characterised by severe neurodevelopmental delays and varying degrees of muscle abnormalities [22]. TRAPPC2L mutations affect its interaction with the core subunit TRAPPC6a and disrupt membrane transport and the function of TRAPP complex. However, whether this complex is also involved in the development of other systems is unknown.

In order to investigate the physiological functions of TRAPP complex, we generated a *Trappc2l* knockout mouse model. Surprisingly, no obvious developmental abnormalities were observed in both adult male and female *Trappc2l* knockout mice. However, the male mice were completely infertile with massive germ cell loss at 2 weeks after birth. By contrast, the germ cell development in female *Trappc2l* ^−/−^ mice was not affected. Further study revealed that deletion of *Trappc2l* caused germ cell syncytial structures formation at E17.5 which in turn caused germ cells death. Further studies found that the syncytial structures were most likely formed by abnormal cell division without cytokinesis. This study demonstrated that *Trappc2l* is involved in male germ cell development and probably by regulating the mitotic arrest of gonocytes during embryonic development.

## 2 Results

### 2.1 Inactivation of *Trappc2l* Caused Germ Cell Loss and Male Infertility

**FIGURE 1:**
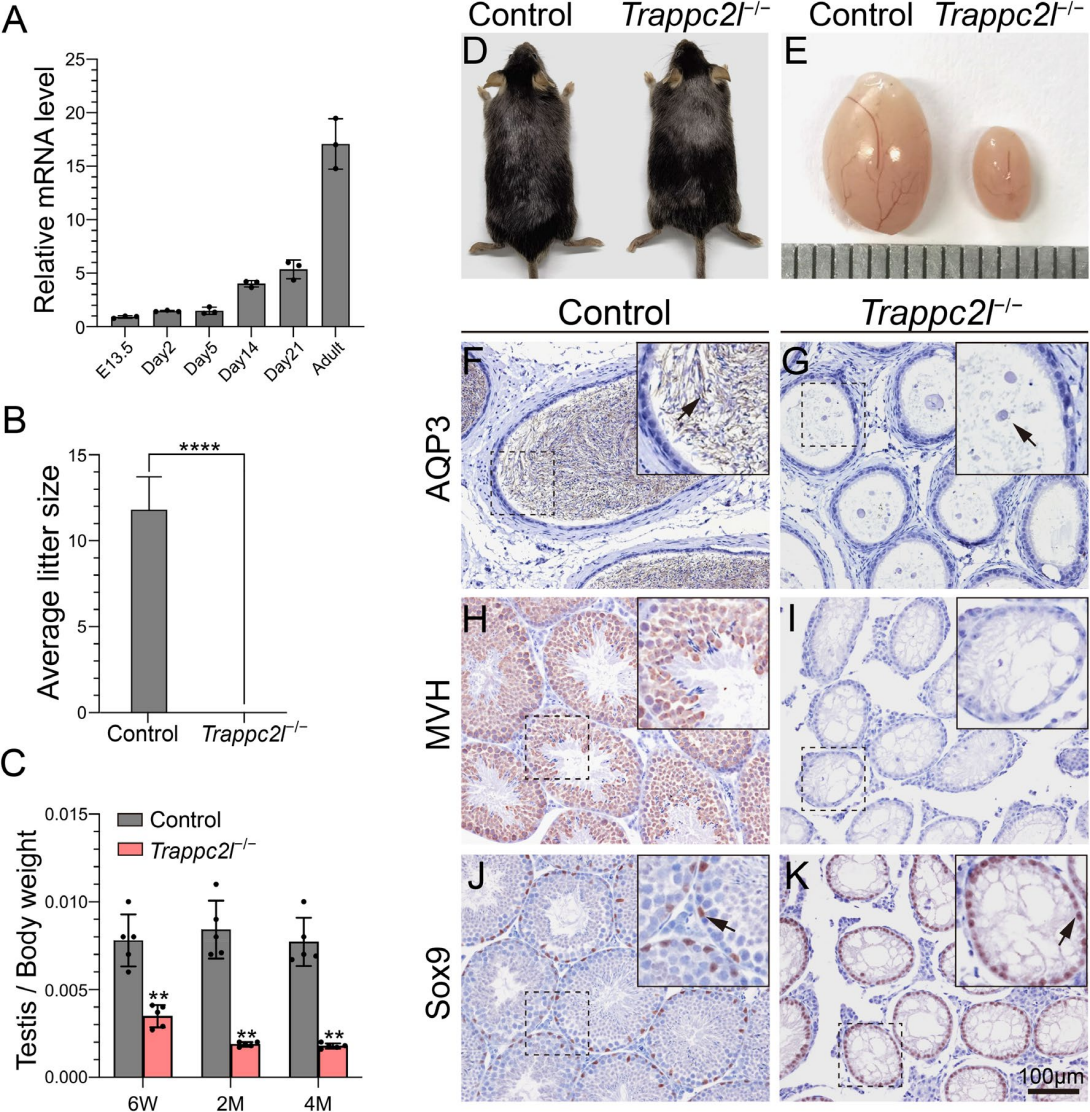
Inactivation of *Trappc2l* causes massive germ cell loss in males. (A) The mRNA level of *Trappc2l* was increased from 2 weeks onward in testes. (B) Fertility test of control and male *Trappc2l* ^−/−^ mice. (C) The ratio of testis/body weight was significantly decreased in *Trappc2l* ^−/−^ mice. (D) Representative photograph of adult control (left side) and *Trappc2l* ^−/−^ (right side) mice. (E) The testes of control (left side) and *Trappc2l* ^−/−^ (right side) mice. A large number of AQP3‐positive sperm tails were observed in the epididymis of control mice (F, black arrow), and no mature sperm but cellular debris and vacuoles were observed in *Trappc2l* ^−/−^ mice (G, black arrow). Germ cells were labelled with MVH (H, brown). No MVH‐positive germ cells were observed in seminiferous tubules of *Trappc2l* ^−/−^ mice (I). The expression of Sertoli cells specific marker gene Sox9 (brown) was detected in both control (J, black arrow) and *Trappc2l* ^−/−^ mice (K, black arrow).

We first examined the mRNA level of *Trappc2l* in mouse testes at different developmental stages by RT‐PCR. As shown in Figure 1A, a relative low level of *Trappc2l* mRNA was detected in testes from E13.5 to Day5. The expression of *Trappc2l* mRNA was increased at Day 14 and high level of *Trappc2l* mRNA was detected in adult testes. To test the functions of *Trappc2l*, we generated *Trappc2l* ^−/−^ mice using CRISPR‐Cas9 technology. As shown in Figure S1A, a truncated mutation was created by deleting exon 2. Genomic PCR confirmed the successful knockdown of *Trappc2l* (Figure S1B). RT‐PCR results showed that *Trappc2l* transcripts were virtually absent in the testes of *Trappc2l* ^−/−^ mice (Figure S1C). Adult *Trappc2l* ^−/−^ mice were grossly normal, and no obvious developmental abnormalities were observed in both male and female mice (Figure 1D). Female *Trappc2l* ^−/−^ mice exhibited normal oogenesis and no defects in fertility were noted (Figure S2). However, *Trappc2l* ^−/−^ male mice were completely infertile (Figure 1B), and the size of testes and testis‐to‐body weight ratio was dramatically reduced compared to the control mice (Figure 1C,E).

We first examined the histology of testes in control and *Trappc2l* ^−/−^ mice at 6 weeks of age. As shown in Figure 1F, a large number of mature sperm were observed in the epididymis of control mice, whereas no sperm was noted in *Trappc2l* ^−/−^ mice (Figure 1G). The results of immunohistochemistry (IHC) showed MVH‐positive germ cells at different developmental stages were observed in seminiferous tubules in control mice (Figure 1H). By contrast, no MVH‐positive germ cells were observed in the seminiferous tubules of *Trappc2l* ^−/−^ mice (Figure 1I). Sox9‐positive Sertoli cells were arranged at the periphery region of seminiferous tubules in both control and *Trappc2l* ^−/−^ mice, (Figure 1J,K). These results indicated that inactivation of *Trappc2l* caused complete germ cell loss which is resemble to the NOA in human patients.

### 2.2 Germ Cell Syncytial Structures Were Observed in *Trappc2l* ^*−/−*^ Mice

**FIGURE 2:**
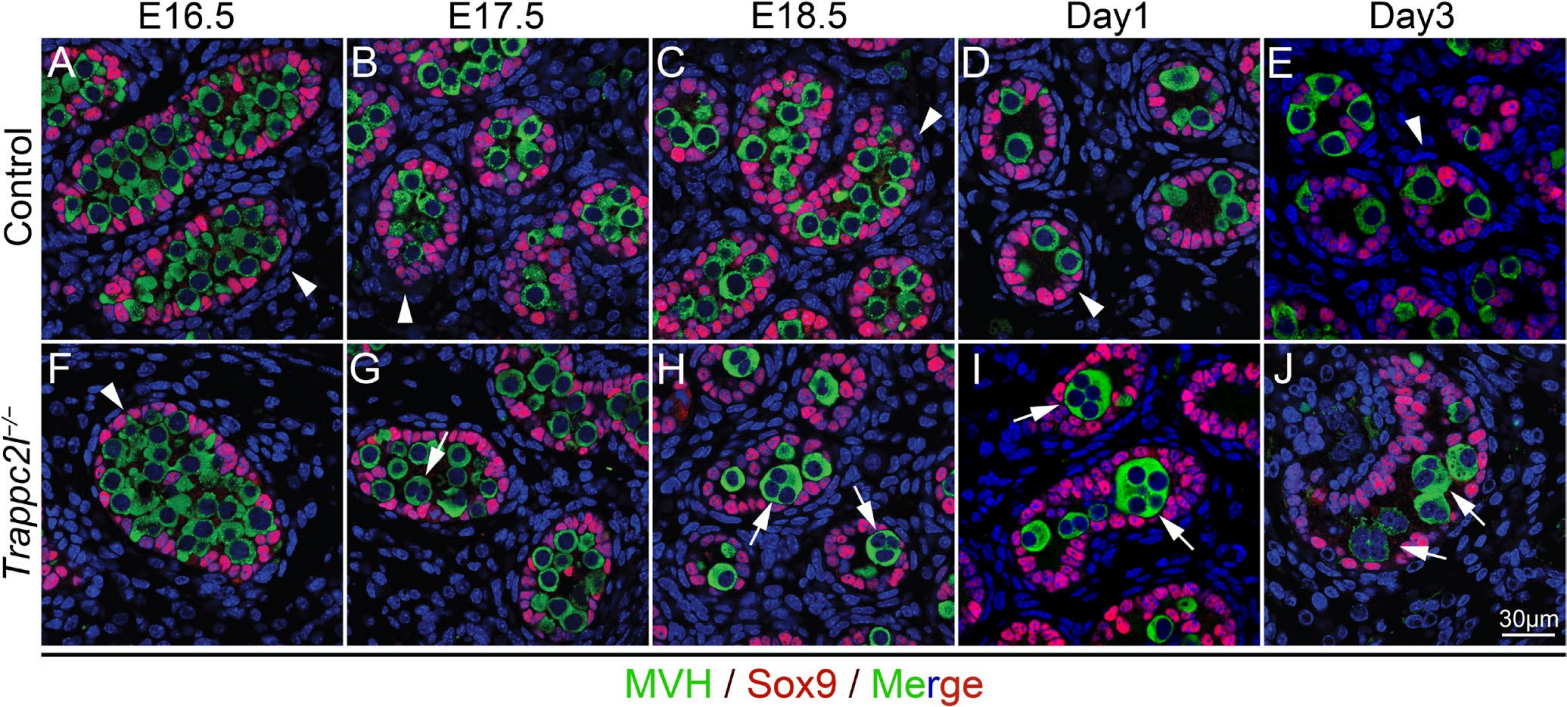
Inactivation of *Trappc2l* resulted in the formation of germ cell syncytial structures. The expression of MVH and Sox9 in control (indicated by white arrowheads) and *Trappc2l* ^*−/−*^ mice was examined by immunohistochemistry. No significant differences were observed at E16.5 between control and *Trappc2l* ^*−/−*^ mice (A,F). MVH‐positive syncytial structures with multiple nuclei (indicated by white arrows) were first observed in *Trappc2l* ^*−/−*^ mice at E17.5 (B,G), and the number of syncytial structures was gradually increased at E18.5 (C,H), day 1 (D,I) and day 4 (E,J) after birth.

To further explore the cause of germ cell loss, we examined the development of germ cells in *Trappc2l* ^*−/−*^ mice at different developmental stages. No obvious abnormalities of germ cell development were noted in *Trappc2l* ^*−/−*^ mice (Figures 2F and S3B) at E16.5 compared to controls (Figures 2A and S3A). A small number of germ cell syncytial structures with multiple nuclear were first observed in the seminiferous tubules of *Trappc2l* ^*−/−*^ mice at E17.5 and E18.5 (Figures 2B,C,G,H and S3C,D). The number of germ cell syncytial structures was gradually increased after birth (Figure 2D,E,I,J). No single germ cell was observed in the seminiferous tubules of *Trappc2l* ^*−/−*^ mice and only germ cell syncytial structures were observed at Day10 (Figure S3E–G). MVH‐positive germ cells were completely absent in the seminiferous tubules of *Trappc2l* ^*−/−*^ mice at 2 weeks after birth (Figure S4A–F). These results indicated that inactivation of *Trappc2l* caused the formation of germ cell syncytial structures which was probably the major reason that led to the gem cell loss in *Trappc2l* ^*−/−*^ mice.

### 2.3 Germ Cell Syncytial Structures in *Trappc2l* ^−/−^ Mice Were Not Formed by Cell Fusion

**FIGURE 3:**
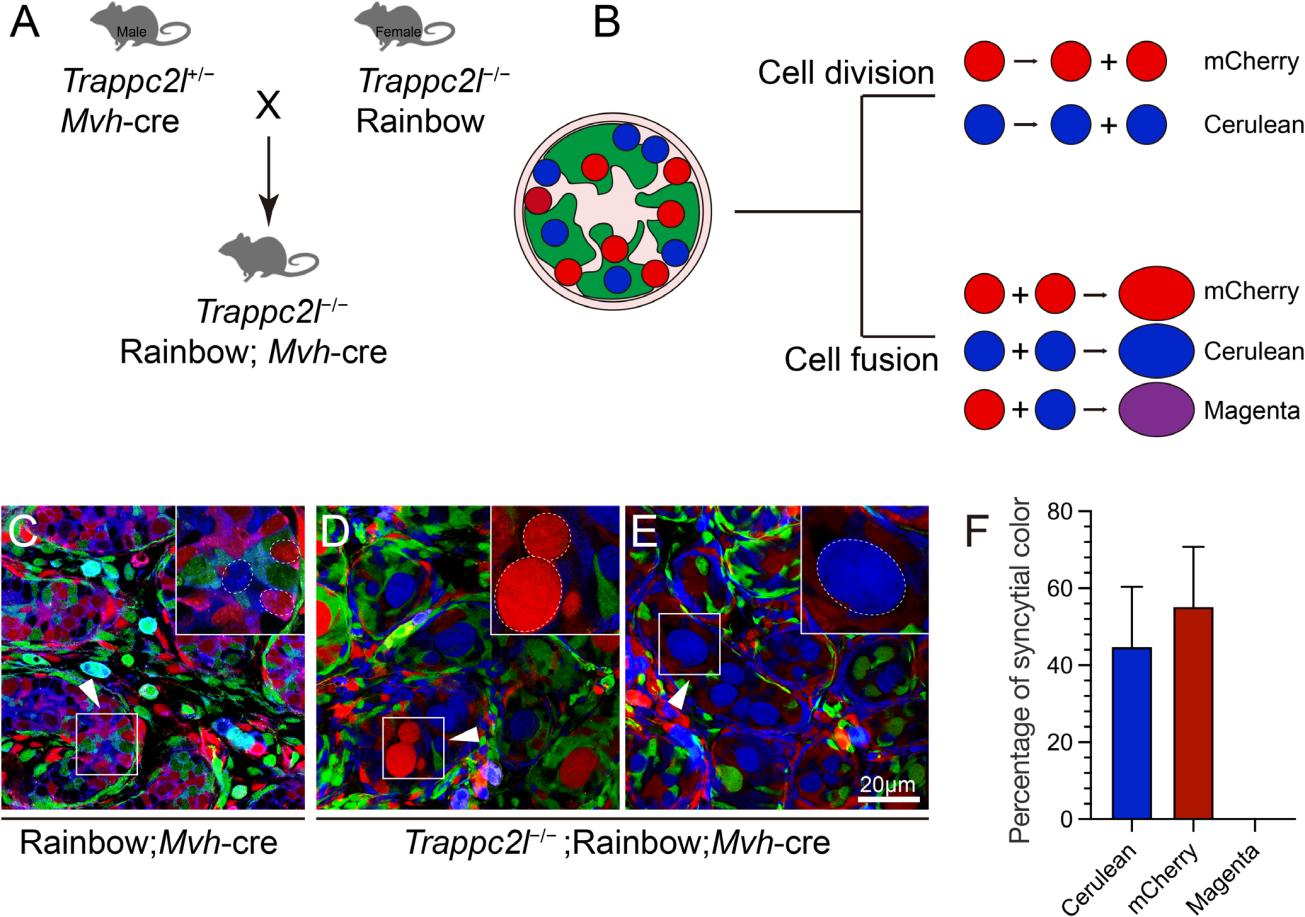
Syncytial structures in *Trappc2l* ^−/−^ were formed by nuclear division without cytokinesis. (A) Strategy for obtaining *Trappc2l* ^−/−^; Rainbow; *Mvh*‐cre mice. (B) A schematic diaphragm of a working model for Rainbow mice. If syncytial structures are formed by incomplete cell division, all syncytia should be labelled with a single fluorescent marker, either Cerulean or mCherry. Conversely, if syncytial structures are raised by cell fusion, the mixed colour magenta should be observed in some syncytial structures. In Rainbow, *Mvh*‐cre mice at day 3, germ cells are randomly labelled with either mCherry or Cerulean (C, white arrowhead). In *Trappc2l* ^−/−^; Rainbow; *Mvh*‐cre mice at day3, syncytial germ cells were also labelled either mCherry (D, white arrowhead) or Cerulean (E, white arrowhead), and no magenta was observed in syncytial structure (F).

**FIGURE 4:**
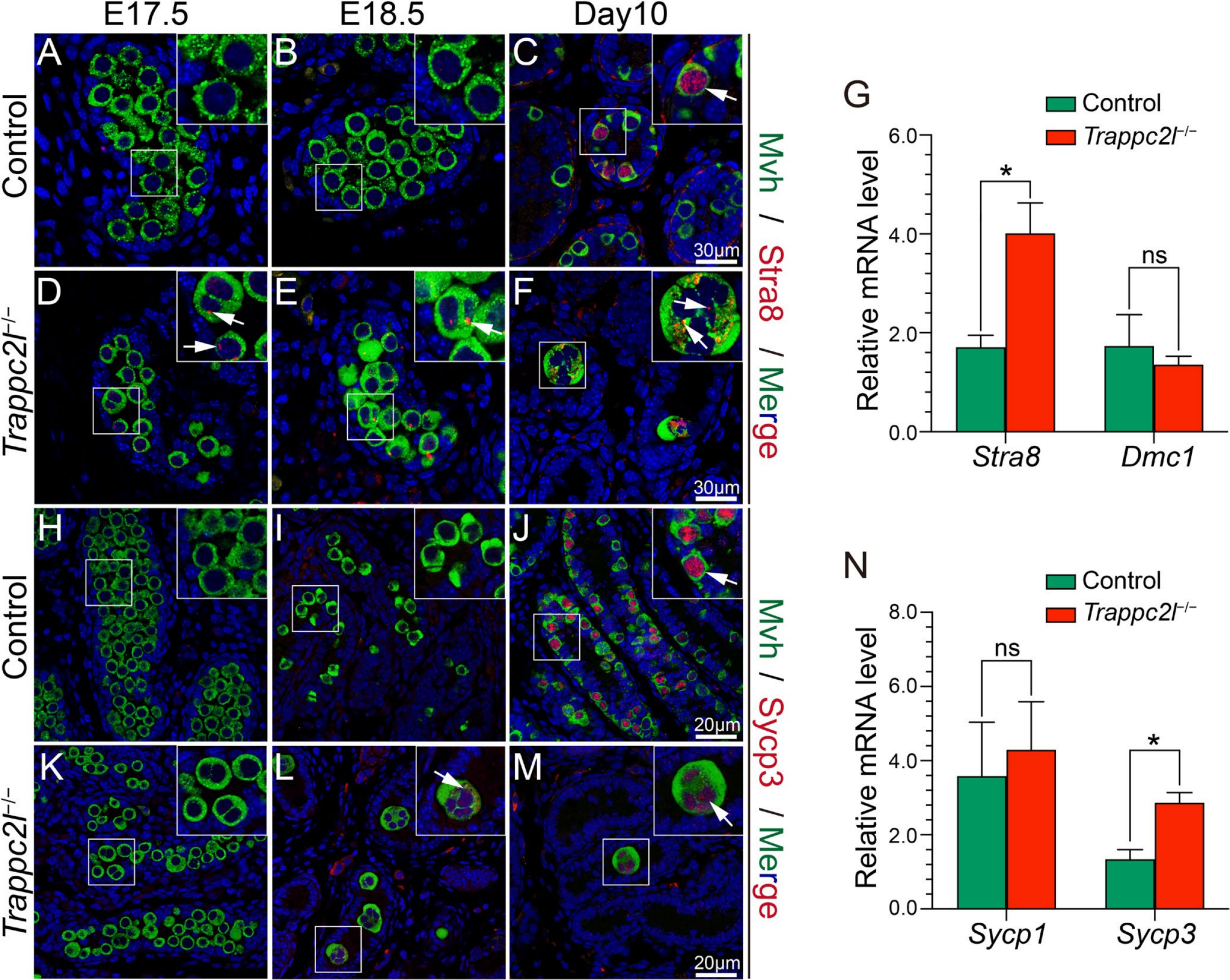
Meiosis‐associated genes were expressed in the germ cells of *Trappc2l* ^*−/−*^ mice at E18.5. (A–M) The expression of Stra8, Sycp3 and Mvh in the testes of control, and *Trappc2l* ^**−/−**^ males was examined by immunofluorescence. No Stra8 was expressed in germ cells of control testes at E17.5 (A) and E18.5 (B), and it was detected in germ cells at D10 (C, white arrow). Very weak signal of Stra8 were detected in the germ cells of *Trappc2l* ^**−/−**^ testes at E17.5 (D, white arrows), E18.5 (E, white arrow) and D10 (F, white arrows). No Sycp3 was expressed in germ cells of control testes at E17.5 (H) and E18.5 (I), and it was detected in germ cells at D10 (J, white arrow). No Sycp3 was expressed in germ cells of *Trappc2l* ^**−/−**^ testes at E17.5 (K). Very weak signal of Sycp3 was detected in the germ cells of *Trappc2l* ^**−/−**^ testes at E18.5 (L, white arrow) and D10 (M, white arrow). The mRNA levels of *Stra8* (G) and *Sycp3* (N) were significantly increased in *Trappc2l* ^**−/−**^ testes compared to control testes at E18.5. However, the levels of *Dmc1* (G) and *Sycp1* (N) were not significantly changed between *Trappc2l* ^**−/−**^ and control testes at E18.5. Data were presented as mean ± SEM. *p < 0.05.

**FIGURE 5:**
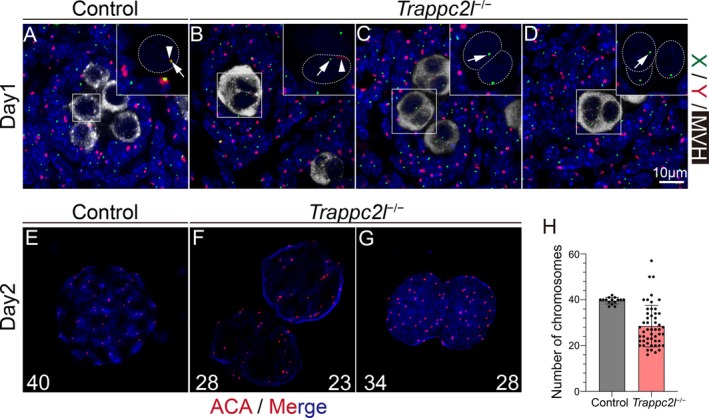
Asymmetric distribution of chromosomal DNA in the nucleus of syncytial structure of *Trappc2l* ^−/−^ mice. The distribution of sex chromosomes in day1 male germ cells of control and *Trappc2l* ^−/−^ mice was assessed using fluorescence in situ hybridization (FISH). The X chromosome was labelled with green DNA probes and the Y chromosome with red DNA probes. In control germ cells, each nucleus contained one X chromosome (A, white arrow) and one Y chromosome (A, white arrowhead). In *Trappc2l* ^−/−^ mice, some nucleus in syncytial cells structure contained one X chromosome (B, white arrow) and one Y chromosome (B, white arrowhead), but numerous nucleus contained only one X chromosome (C, D white arrow). The expression of anti‐centromere antibody (ACA) was examined by immunofluorescence. In control mice, 40 ACA‐positive loci were observed in the nucleus of germ cells (E). By contrast, the number of ACA‐positive loci was inconsistent in the nucleus of germ cells syncytial cells structure of *Trappc2l* ^−/−^ mice (F,G). Number of chromosomes was mostly between 20 and 40 (H).

To explore the reason which caused germ cell syncytial structure formation in *Trappc2l* ^*−/−*^ mice, we first labelled the cell membranes with Na/K‐ATPase antibodies to assess membrane integrity. As shown in Figure S5A–H, the Na/K‐ATPase signal was lined at the outer layer of the syncytial structures and the signal was continuous, indicating that these structures were intact syncytium. There are two possible reasons for the formation of syncytial structures. The first one is that germ cells were fused together after inactivation of *Trappc2l*. The second one is abnormal cell division with incomplete cytokinesis. To test whether the syncytial structure is formed by germ cell fusion, we used Rainbow mouse model to label the germ cell with different fluorescent. Rainbow mice were crossed with *Trappc2l* ^*−/−*^ and MVH‐Cre mice to generate *Trappc2l* ^*−/−*^; Rainbow and MVH‐Cre mice (Figure 3A). In this model, germ cells were randomly labelled with mCherry or Cerulean fluorescent under the induction of MVH‐Cre. If the germ cells were fused in *Trappc2l* ^*−/−*^ mice, the mixed colours (Magenta) would be observed in some syncytia. As shown in Figure 3B,C, germ cells were randomly labelled with red (mCherry) or blue (Cerulean) fluorescence in control mice (Rainbow; MVH‐Cre). In *Trappc2l* ^*−/−*^ mice, all the germ cell syncytia were labelled with either mCherry or Cerulean fluorescence, and no mixed colour (Magenta) labelled syncytia were observed (Figures 3B,D–F and S6). These results indicated that germ cell syncytial structures were not formed by cell fusion. The second possible reason is abnormal cell division with incomplete cytokinesis. To test cell proliferation in *Trappc2l* ^*−/−*^ mice, we first analysed the expression of mitotic genes Ki67 and PH3 by immunostaining (Figure S7A–P). Ki67 signals were detected in Sertoli cells of both control and *Trappc2l* ^*−/−*^ testes (Figure S7I–P). By contrast, no Ki67 or PH3 positive signal was detected in germ cells of control mice and syncytial structures of *Trappc2l* ^*−/−*^ mice at E17.5‐Day1. At around postnatal day 3, positive signals for PH3 and KI67 were detected in some germ cells of the control mice (Figure S7D,L white arrow), while no positive signals for PH3 or KI67 were detected in the syncytial structures of the age‐matched *Trappc2l* ^*−/−*^ mice (Figure S7H,P). Subsequently, we used BrdU to assess DNA replication in germ cells (Figure S7Q–T). BrdU‐labelled Sertoli cells were detected in both control and *Trappc2l* ^*−/−*^ mice at E17.5 and Day 1. By contrast, no BrdU signal was detected in germ cells of control mice and the syncytial structures in *Trappc2l* ^*−/−*^ mice. The results of RT‐PCR showed that the mRNA levels of proliferation‐related genes, including Mcm2, PCNA and Ki67, were not changed in the testes of *Trappc2l* ^*−/−*^ mice compared to the control testes at E18.5 (Figure S7U). These results suggest that the gonocytes in *Trappc2l* ^*−/−*^ mice did not undergo the classical DNA replication process. It has been demonstrated that the male germ cells undergo meiosis arrest during the embryonic stage. To test whether the inactivation of *Trappc2l* caused abnormal initiation of meiosis during embryonic which led to the formation of syncytial structures, we first examined the expression of meiosis‐related genes. As shown in Figure 4A,B,H,I, no expression of STRA8 and SYCP3 was detected in germ cells of control testes at E17.5 and E18.5. By contrast, a very weak signal of STRA8 or SYCP3 was detected in germ cells of *Trappc2l* ^*−/−*^ mice at E17.5 (Figure 4D) and E18.5 (Figure 4E,L). No expression of SYCP3 was detected in germ cells of *Trappc2l* ^*−/−*^ mice at E17.5 (Figure 4K). At around postnatal day 10, germ cell nuclei in the control group began to normally express STRA8 and SYCP3 (Figure 4C,J), while in the germ cells syncytial structures of the age‐matched *Trappc2l* ^*−/−*^ mice, the expression of STRA8 and SYCP3 remained weak (Figure 4F,M). The mRNA levels of STRA8 and SYCP3 were significantly increased in *Trappc2l* ^*−/−*^ testes compared to control testes at E18.5 (Figure 4G–N). However, the levels of *Dmc1* and *Sycp1* were not significantly changed between *Trappc2l* ^*−/−*^ and control testes. To further confirm whether the premature expression of STRA8 and SYCP3 led to the abnormal meiosis, chromosome ploidy and karyotype of nuclear in syncytial structures were examined by FISH analysis and immunostaining of anti‐centromere antibody (ACA) with chromosome spread. FISH analysis showed that each germ cell in control mice contained one X and one Y chromosome (Figure 5A). In the syncytial structures from *Trappc2l* ^*−/−*^ mice, we observed nuclei containing both X and Y chromosomes (Figure 5B), as well as nuclei with only a single chromosome (Figure 5C,D). To examine the karyotype of germ cell syncytial structures, chromosome spread was prepared with germ cells from control and *Trappc2l* ^*−/−*^ mice at postnatal day 2 and centromeres were labelled with ACA. The results of immunofluorescence (IF) showed that germ cells in control mice exhibited the normal count of 40 chromosomes (Figure 5E), while the number of chromosomes in the nuclear of germ cells syncytial structures was inconsistent, most of which were between 20 and 40 (Figure 5F–H). Based on these results, we concluded that the syncytial structures in *Trappc2l* ^*−/−*^ mice were not caused by abnormal initiation of meiosis. The germ cell syncytial structures were formed probably by abnormal cytokinesis without DNA replication.

### 2.4 The Aberrant Germ Cell Development in *Trappc2l* ^−/−^ Mice Was Caused by Defects in Sertoli Cells

To explore whether the loss of germ cells in *Trappc2l* ^−/−^ mice was caused by defects in Sertoli cells development, we first examined the expression of TRAPPC2L in testis. Because no commercially available antibodies could be used for immunostaining. We generated an EGFP‐tagged *Trappc2l* knock‐in mouse model (Figure S8A) utilising CRISPR/Cas9 system. The mouse model was verified by western blot analysis (Figure S8B). To determine whether the functions of the TRAPPC2L are affected by fusion with the EGFP tag, we first assessed the fertility of homozygous *Trappc2l*‐EGFP mice and found that homozygous *Trappc2l*‐EGFP male mice were fertile (Figure S8C). These results indicated that the EGFP tag did not affect the functions of the TRAPPC2L. The immunostaining results showed punctate GFP signals were detected in the cytoplasm of both Sertoli cells (Figure S9E–L, white arrows) and germ cells (Figure S9E–L, white arrowheads), while no GFP signal was detected in control testis (Figure S9A–D).

To further confirm the functions of *Trappc2l* in Sertoli cells, GFP‐positive control SSCs were transplanted into the seminiferous tubules of *Trappc2l* ^−/−^ mice. The development of transplanted germ cells was examined at 1 month post‐transplantation. As shown in Figure S10A,B, GFP‐positive SSCs were successfully colonised in the seminiferous tubules of *Trappc2l* ^−/−^ mice, and germ cells at various developmental stages were observed. In contrast, no germ cells were detected in *Trappc2l* ^−/−^ mice without SSCs injection. To further clarify the cell‐specific role of *Trappc2l* in spermatogenesis, we generated a *Trappc2l* conditional knockout model and selectively inactivated *Trappc2l* in somatic cells by crossing with *Sf1*‐Cre mice. We found that the development of germ cell in adult *Trappc2l* ^f/f^; *Sf1*‐Cre mice was not affected (Figure S11A,B). These results indicated that *Trappc2l* plays cell autonomous functions in germ cell development.

## 3 Discussion

TRAPPC2L is a highly conserved member of the TRAPP II and TRAPP III complexes [13, 21]. As a core subunit of the TRAPP II and TRAPP III complexes, TRAPPC2L plays a crucial role in vesicle transport from the ER to the Golgi apparatus and in the later stages of the secretory pathway. It has been reported that a homozygous missense mutation in TRAPPC2L gene was detected in two unrelated patients with neurodevelopmental delay and epilepsy. Furthermore, whole‐genome sequencing of three siblings with developmental delay and intellectual disability revealed a homozygous missense mutation in TRAPPC2L [23]. Other than brain, TRAPPC2L is also highly expressed in testicular tissue. However, the function of TRAPPC2L in the reproductive system has not been studied yet.

In this study, we found that *Trappc12* knockout mice are grossly normal but the male mice were infertile and all the germ cells were lost due to the formation of germ cell syncytial structures. We also demonstrated that the syncytial structures were formed by aberrant cell division with incomplete cytokinesis. In mammals, multinucleated cells are widely observed under normal physiological conditions, particularly in skeletal muscle and the placenta. In skeletal muscle, cells form multinucleated structures that allow for efficient protein synthesis and metabolic activity to meet the demands of intense muscular function [24, 25]. In the placenta, syncytiotrophoblast cells adopt a similar multinucleated morphology, which provides an enlarged cell surface area to better support material exchange between the mother and fetus [26]. Under pathological conditions, such as in patients with cryptorchidism, multinucleated cells may also be present [27, 28]. These cells may arise due to the ineffective clearance of senescent or degenerated cells within the seminiferous tubules. Multinucleated cells associated with cryptorchidism are potentially involved in compensatory or clearance functions and are linked to mechanisms of apoptosis and tissue repair, although the specific mechanisms underlying their formation remain unclear [29]. Previous studies have demonstrated that the meiotic arrest of male germ cell during the embryonic stage is due to the lack of RA [30, 31, 32]. However, the mechanisms which regulate mitotic arrest during the embryonic stage are still unknown. In this study, we found that the inactivation of *Trappc12* gene caused aberrant cell division of germ cell during the embryonic stage. However, the cytokinesis was incomplete and DNA replication was not detected. This phenomenon is similar to a division mode called ‘asynthetic fission’ which is originally observed in superficial epithelial cells (SECs) of zebrafish larvae to meet rapid coverage needs, but it has been rarely studied in mammals [33]. Intriguingly, multi‐nuclear was observed in the germ cell syncytial structures and the karyotype in the nuclear syncytial structures is abnormal. The number of chromosomes was between 20 and 40 in most of the nuclear. The results of FISH analysis showed that some unclear syncytial structures contained a single X or Y chromosome, but most of the unclear still contained both X and Y chromosomes. These results indicated that the chromosomes were asymmetrically separated during division. Although no cell fusion was observed and the expression of mitotic‐related genes was not detected in the germ cells of *Trappc12* mice, multiple nuclear were found and the total number of chromosomes in the syncytial structures was more than 40. These results suggested that the DNA probably replicated via other unknown mechanisms which need further investigation.

The TRAPPC complex is a multi‐subunit complex that is involved in various physiological processes such as vesicle transport, autophagy and glycosylation [34]. In addition to its role in membrane trafficking between the ER and the Golgi apparatus, the TRAPP complex also plays a crucial role in the cell cycle, particularly during mitosis and the later stages of cell division [35]. During cytokinesis, a substantial amount of new membrane formation is required at the cleavage furrow region. TRAPP‐II complexes are involved in anchoring exocytic vesicles, which carry new membrane material, to the cleavage furrow, ensuring the accurate deposition of the new membrane [36, 37]. Previous studies have demonstrated that depletion of *Trappc12* impedes the recruitment of the kinetochore protein CENP‐E and disrupts proper chromosome congression which causes mitotic arrest [38, 39]. Based on these studies, we speculated that TRAPP complex‐mediated vesicle transfer probably plays important roles in regulating the mitotic arrest of male germ cell during the embryonic stage. However, the primary functions of *Trappc2l* are to assist vesicle recognition and fusion with target membranes rather than directly interact with cargo proteins. Traditional immunoprecipitation assays cannot directly capture the cargo proteins. Proximity labelling techniques are probably need to be used in the future.

In summary, in this study, we found that TRAPPC2L plays critical roles in male germ cell development. Inactivation of *Trappc2l* caused male germ cells abnormal cell division and formed syncytial structures which in turn led to germ cell loss and male infertility. The results of our study suggest that TRAPP complex‐mediated vesicle transfer probably plays important roles in regulating the mitotic arrest of male germ cell during the embryonic stage which will shed light on a better understanding of the regulation of germ cell development during the embryonic stage.

## 4 Materials and Methods Animals

All animal experiments were carried out according to the protocols approved by the Institutional Animal Care and Use Committee (IACUC) of the Institute of Zoology, Chinese Academy of Sciences (CAS; SYXK 2018–0017). All mice were maintained on a C57BL/6;ICR;129/SvEv mixed background and housed in specific pathogen‐free (SPF) conditions under a 12 h light–dark cycle with a room temperature of 20°C–24°C and humidity of 35% ± 4%. *Trappc2l* knockout mice were generated using the CRISPR‐Cas9 system by Cyagen Biosciences. *Trappc2l* knockout mice were generated using the CRISPR‐Cas9 system by the Animal Experiment Center, Institute of Zoology. The primers used for genotyping were listed in Table S1.

### 4.1 Rt‐PCR

Total RNA was extracted using the RNeasy Kit in accordance with the manufacturer's instructions. 1 μg of total RNA was used to synthesise first‐strand cDNA (Abm, G592). cDNAs were diluted and used for the template for real‐time SYBR Green assay. Gapdh was used as an endogenous control. All gene expression was quantified relative to Gapdh expression. The relative expression level of candidate genes was calculated using the formula 2^−ΔΔCT^. The primers for *Trappc2l* were summarised in Table S1.

### 4.2 Histological Analysis

Tissues were fixed in 4% paraformaldehyde (PFA) for up to 24 h, stored in 70% ethanol and embedded in paraffin. Five‐micrometre‐thick sections were prepared and mounted on glass slides.

After deparaffinisation, slides were stained with H&E for histologic analyses.

### 4.3 Periodic Acid–Schiff (PAS) Staining

PAS staining was performed as previously described (Sun and Handel, 2011) with modification. 5‐μm paraffin sections of mouse testes were dewaxed and treated with 0.5% periodic acid for 30 min and Schiff's reagent for 90 min. After being washed three times with distilled water, slides were then stained with haematoxylin and sealed with neutral gum.

### 4.4 Immunohistochemistry and IF Analysis

IHC was performed as previously described (57). Paraffin sections of the testes were incubated with the primary antibodies for 1 h. After washing with phosphate‐buffered saline (PBS), the slides were incubated with secondary antibodies. Slides were finally examined with confocal microscopy (Nikon, Japan), Images were captured by a Nikon DS‐ Ri1 CCD camera. The information for the antibodies was summarised in Table S2.

### 4.5 Chromosome Spread and IF

Testes were decapsulated and incubated with hypotonic extraction buffer (HEB; 30 mM Tris, pH 8.2, 50 mM sucrose, 17 mM trisodium citrate dihydrate, 5 mM EDTA, 0.5 mM DTT and 0.5 mM PMSF) for 45 min at room temperature. The seminiferous tubules were incubated with 100 μL sucrose (100 mM) after the removal of the hypotonic extraction buffer. The seminiferous tubules were torn into small pieces with tweezers and pipetted 10 μL cell suspension onto the slides covered with 500 μL 0.15% Triton X‐100 in 1% PFA (pH 9.2). Slides were placed in a humid chamber and kept for at least 6 h at room temperature. After air dry, the slides were stored at −80°C for further experiments.

Slides were incubated with 0.4% Kodak Photo‐Flo 200 in water for 4 min. After washing with 1 PBST (0.1% Triton X‐100 in PBS) for three times, the slides were blocked with 200 μL blocking buffer (3% non‐fat milk in 1 PBST) for 1 h at room temperature. After incubating with primary antibodies (3% non‐fat milk buffer) overnight at 4°C. Slides were washed with 1 PBST (0.1% Triton X‐100 in PBS) for three times and incubated with fluorochrome‐conjugated secondary antibodies in the dark for 1 h at 37°C.

### 4.6 Slide Preparation, Fixation of Mouse Testis and FISH

Mouse testes were surgically removed, fixed in 4% PFA and embedded in paraffin. Sections were cut at a thickness of 5 μm, deparaffinised with xylene and incubated at 98°C for 50 minutes with Histo VT One (Nacalai Tesque, Kyoto, Japan). To observe the signals of gonocytes, the samples were incubated in a solution containing 0.001% proteinase K/PBS at 37°C for 5 minutes. After washing, sections were dehydrated in 70% and 100% ethanol for 5 minutes each and air‐dried. The samples were then incubated with 10 μL of mouse XY chromosome FISH probes (Y: fluorescein isothiocyanate [FITC]; X: biotin; MXY‐10, Chromosome Science Labo, Sapporo, Japan). Slides were covered with parafilm and incubated at 90°C for 10 min on a heat plate. After hybridization in a humid chamber at 37°C for 24 hours, the parafilm was gently removed, and the slides were incubated in 2× standard saline citrate (SSC, Nacalai Tesque, Kyoto). The slides were rinsed in 1× SSC at 37°C for 20 min. Some sections were incubated in Blocking One (Nacalai Tesque) at 4°C for 1 hour, followed by sequential reactions with Alexa Fluor 555 streptavidin (diluted 1:1000, S32355, Invitrogen, Waltham, MA, USA), Hoechst 33342 (diluted 1:5000, Molecular Probes, Eugene, OR, USA), MVH antibody (diluted 1:200, abcam 13840) and Alexa Fluor 647‐labelled anti‐rabbit antibody as the secondary antibody for anti‐MVH. FISH images were obtained by sequential scanning using an LSM‐880 confocal laser microscope.

### 4.7 Western Blotting

Tissue and cell were washed with PBS, lysed with RIPA buffer (50 mM Tris–HCl [pH 7.5], 150 mM NaCl, 1% NP‐ 40, 0.1% SDS, 1% sodium deoxycholate, 5 mM EDTA) supplemented with protease inhibitors cocktail (Roche) and 1 mM PMSF. Equal amounts of total protein were electrophoresed using 10% SDS/PAGE gels. After electrophoresis, the proteins were transferred to a nitrocellulose membrane and probed with the primary antibodies. The images were captured with the ODYSSEY Sa Infrared Imaging System (LI‐COR Biosciences, Lincoln, NE). Densitometry was performed using ImageJ software. The protein level was normalised to that of GAPDH.

### 4.8 Statistical Analysis

All experiments were repeated at least three times. 3–5 mice for each genotype at each time point were used for immunostaining or quantitative experiments. For immunostaining, one representative picture of similar results from 3 to 5 mice for each genotype at each time point was presented. The quantitative results were presented as the mean ± SEM. Statistical analyses were conducted using GraphPad Prism version 9.0.0. Unpaired two‐tailed Student's t‐tests were used for comparison between the two groups. For three or more groups, data were analysed using one‐way. ANOVA. *p*‐Values < 0.05 were considered to indicate significance.

## Author Contributions

M.W. performed the experiments and analysed the data. M.W. wrote the manuscript with assistance from the other authors. All co‐authors critically read and approved the manuscript.

## Ethics Statement

All animal experiments were carried out in accordance with institutional animal care and use committee regulations of the Institute of Zoology, CAS.

## Consent

All authors approved the final manuscript and submission to this journal.

## Conflicts of Interest

The authors declare no conflicts of interest.

## Supporting information


**Figures S1–S11.** Supporting Information.

**Supplementary Table S1.** List of primers used in this study.

**Supplementary Table S2.** List of antibodies used in this study.

## Data Availability

The data that support the findings of this study are available from the corresponding author upon reasonable request.
