# Supplementary material for: TRAPPC2l Participates in Male Germ Cell Development by Regulating Cell Division
Source: Cell Prolif. 2025 Jan 26;58(6):e13810. doi: 10.1111/cpr.13810 (PMC12179553; doi:10.1111/cpr.13810)
Supplement: Supplementary file 1 — Figures S1–S11. Supporting Information. [file CPR-58-e13810-s001.docx]

**Supplementary Figures Legend**


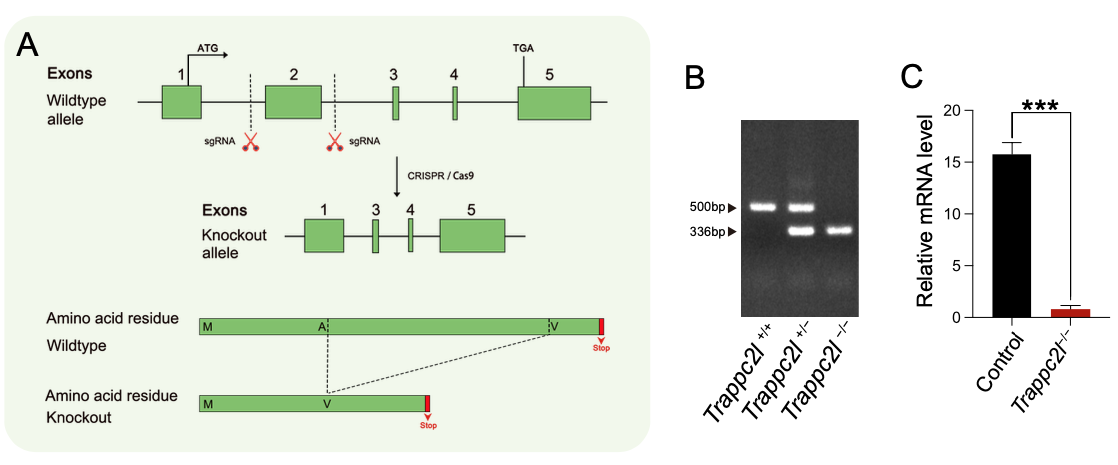


**Figure. S1. Generation of *Trappc2l* knockout mouse model.**

A. Schematic diaphragm of gene targeting strategy used to generate *Trappc2l*^−/−^ mouse model. B. Genotyping of *Trappc2l*^−/−^ mice. C. The mRNA level of *Trappc2l* gene in *Trappc2l*^−/−^ mice was significantly decreased compared to the control mice. Data are presented as mean ± SEM. *P <0.05; **P <0.01; ***P <0.001.


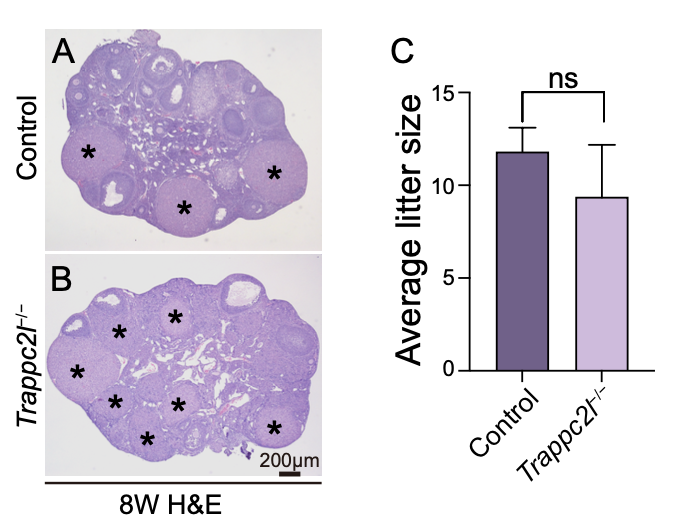


**Figure. S2. Normal follicles development in female *Trappc2l^−/−^* mice.**

The results of H&E staining showed no significant differences in ovarian structure between the control (A) and *Trappc2l*^−/−^ (B) mice at 8W. The corpus luteum were indicated by black asterisks. The average litter size of control and *Trappc2l*^−/−^ female mice at 2 months (n=5) (C). Data are presented as the mean ± SD.


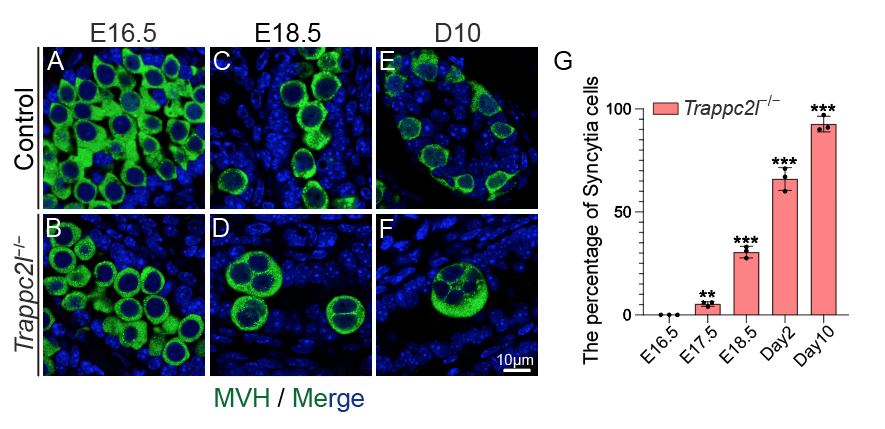


**Figure. S3.** **Inactivation of *Trappc2l* caused germ cells syncytium formation.**

MVH-positive (green) germ cells were observed in both control (A, C, E) and *Trappc2l*^−/−^ (B, D, F) testes at E16.5, E18.5, D10. The percentage of MVH-positive syncytia cells with multiple nuclear was significantly increased from E17.5 to Day10 in *Trappc2l*^−/−^ mice (G). All data are presented as the mean ± SEM. *P < 0.05; **P < 0.01; ***P < 0.001.


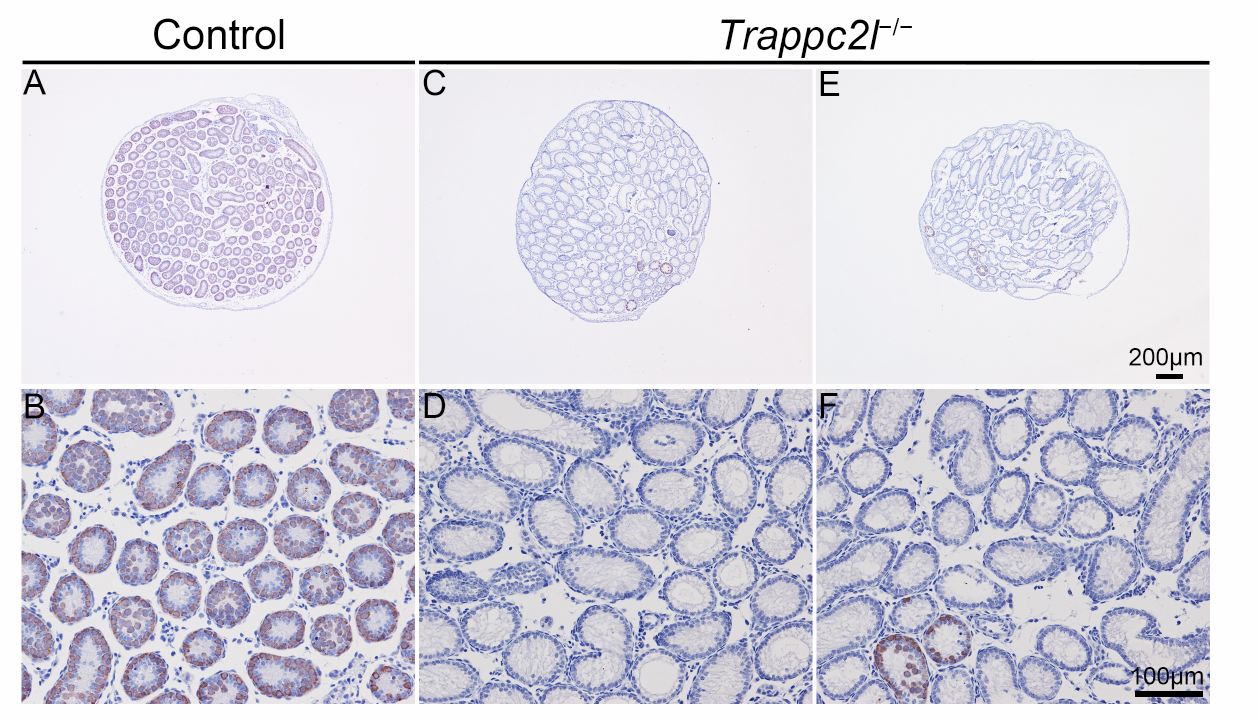


**Figure. S4.** **MVH-positive germ cells were absent in the seminiferous tubules of *Trappc2l*^−/−^ mice at 2 weeks after birth.**

MVH-positive (brown) germ cells were observed in the 2 weeks testes of control mice (A, B). In contrast, very few MVH-positive (brown) germ cells were observed in the testes of 2-week-old *Trappc2l*^−/−^ mice (C-F).


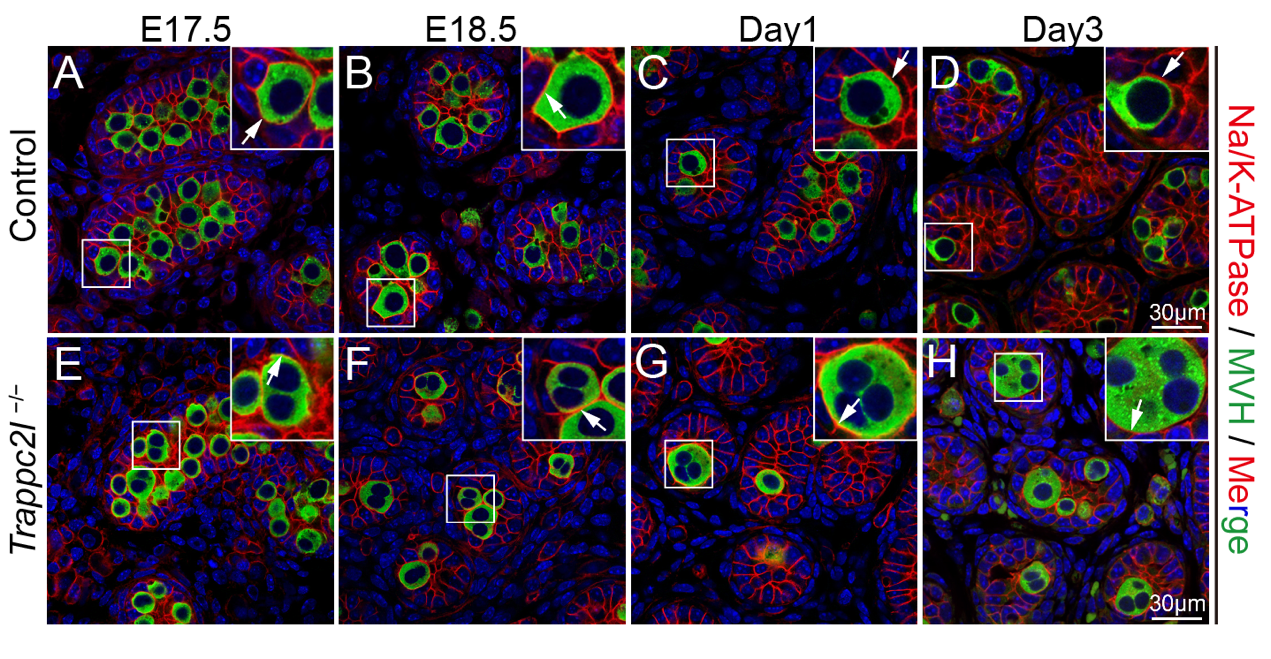


**Figure. S5. The cell membrane of syncytial structures in *Trappc2l*^−/−^ mice were intact.**

The expressions of the cell membrane marker Na/K-ATPase and the germ cell marker MVH in the gonads of control and *Trappc2l^−/−^* mice were examined by immunofluorescence. In control mice, Na/K-ATPase signal was located at the cell membrane of germ cells (A-D, white arrow). The Na/K-ATPase signal was lined at the outer layer of the syncytial structures in *Trappc2l^−/−^* mice (E-H, white arrow) and signal was continuous.


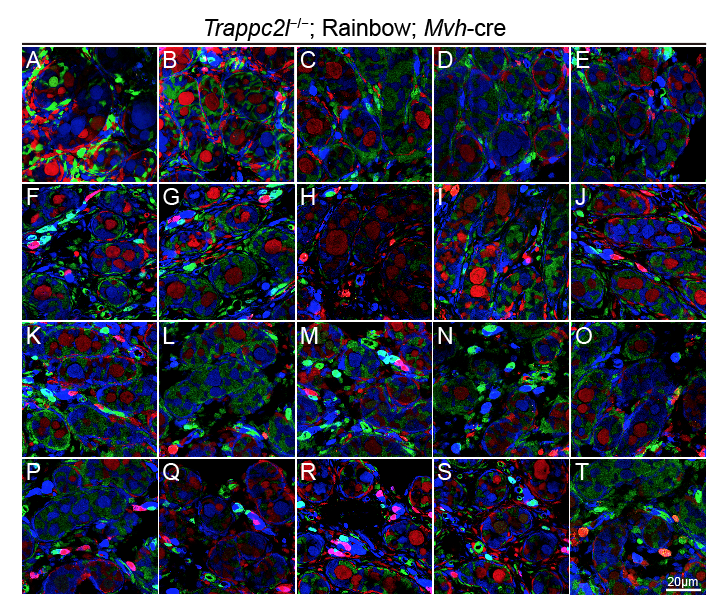


**Figure. S6. Syncytial structures in *Trappc2l^−/−^* were not formed by germ cells fusion.**

In *Trappc2l*^−/−^; Rainbow; *Mvh*-cre mice, all the germ cell syncytia were label with either mCherry or Cerulean fluorescence, no mixed color (Magenta) labeled syncytia were observed

**
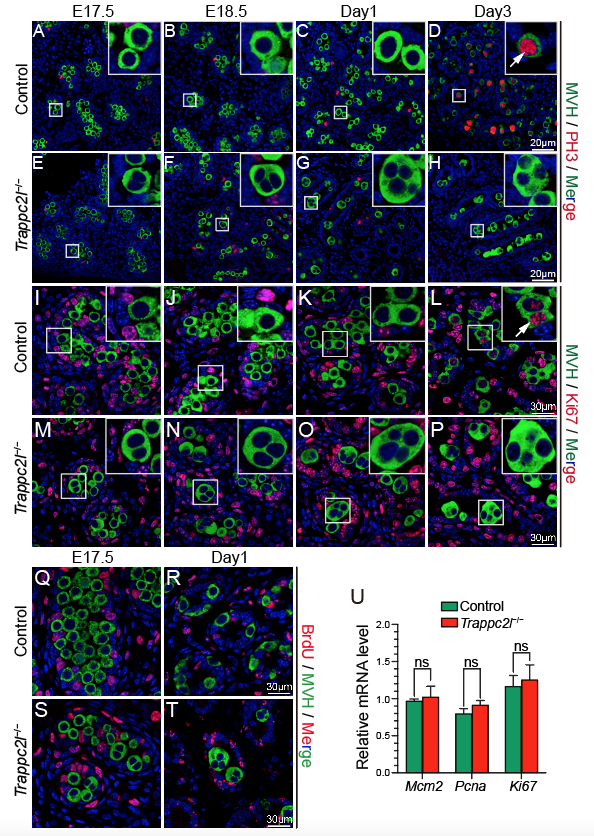
**

**Figure. S7. No proliferative markers and DNA replication were observed in germ cells of *Trappc2l*^−/−^ mice.**

The expression of PH3 and MVH (A-H), Ki67 and MVH (I-P), BrdU and MVH (Q-U) in gonads of control and *Trappc2l***^−/−^** mice was examined by immunofluorescence. No PH3 and Ki67 signal was detected in the MVH positive germ cells and syncytial structures in both control (A-C, I-K) and *Trappc2l^−/−^* (E-G, M-O) mice at E17.5, E18.5, day1 after birth. PH3 and Ki67 signal was detected in the MVH positive germ cells in control at day3 after birth (D, L, white arrow). However, no PH3 or Ki67 signal was detected in the MVH positive syncytial structures in *Trappc2l^−/−^* mice at day3 after birth. No BrdU signal was detected in the MVH positive germ cells and syncytial structures in both control and *Trappc2l^−/−^* mice at E17.5, or day1 after birth (Q-T). The mRNA levels of cell proliferation specific genes, including Mcm2, PCNA and Ki67, were not significantly altered in *Trappc2l***^−/−^** E18.5 testes (U). Data are presented as mean ± SEM.


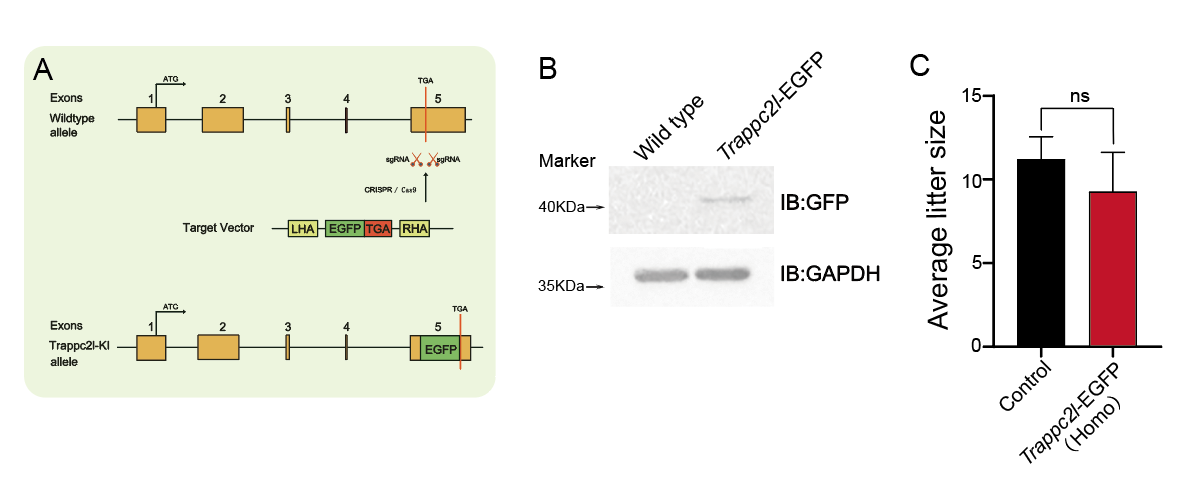


**Figure. S8. Generation of *Trappc2l*-EGFP knock-in mouse model.**

A. Schematic of gene targeting strategy used to generate *Trappc2l*-EGFP mouse model. B. Western blot of *Trappc2l*-EGFP and wild type mice. In *Trappc2l*-EGFP mice, a ~40 kDa band was detected using a GFP antibody, matching the predicted molecular weight of the *Trappc2l*-EGFP fusion protein. C. Fertility test of control and homozygous *Trappc2l*-EGFP male mice.


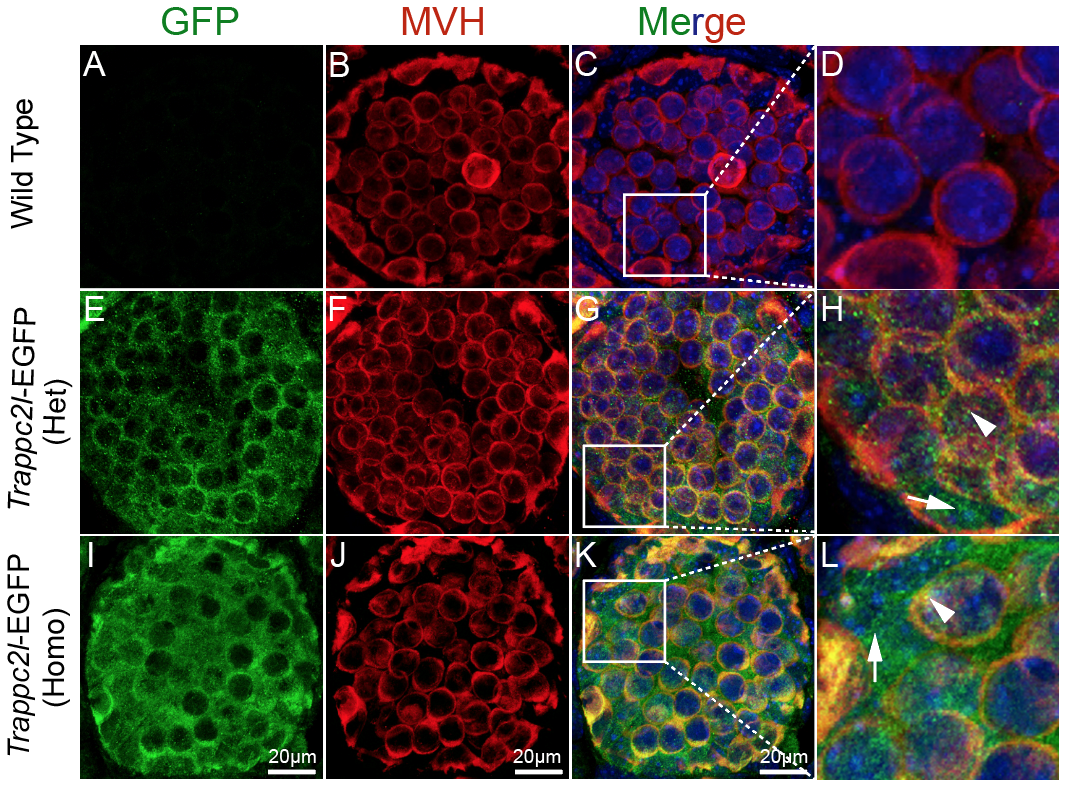


**Figure. S9. TRAPPC2L was expressed in both germ cells and Sertoli cells.**

A–L. Co-immunofluorescence of GFP (green) with MVH (red) in testes of wild type and *Trappc2l*-GFP mice at 2 weeks. No GFP-positive signal was detected in wild-type mice (D), whereas GFP signals were observed in MVH-positive germ cells (white arrowheads) and Sertoli cells (white arrows) in *Trappc2l*-EGFP heterozygous (H) and homozygous mice (L).

**
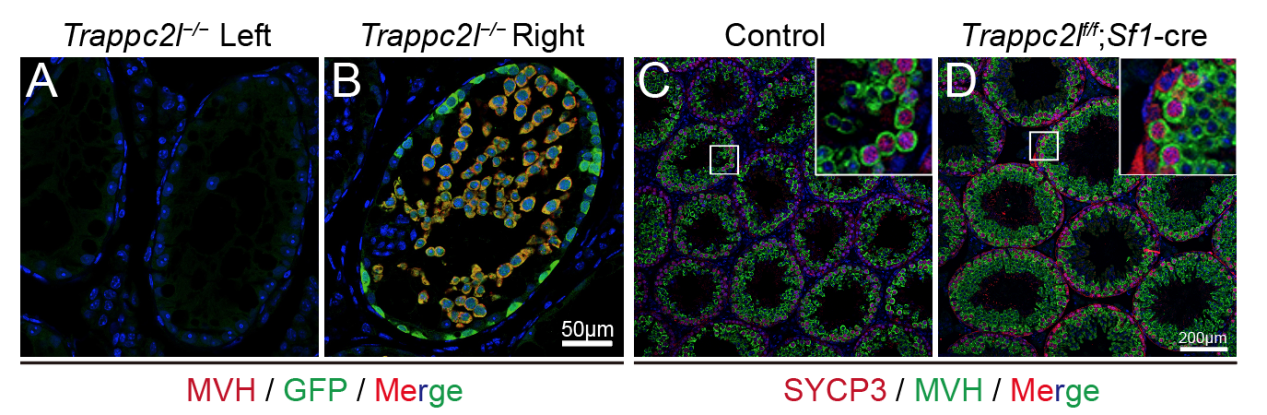
**

**Figure. S10.** **The Sertoli cells of *Trappc2l*^−/−^ mice are capable of supporting spermatogenesis.**

A. No MVH positive germ cells were detected in testes of *Trappc2l*^−/−^ mice without spermatogonia stem cells (SSCs) transplantation. B. Germ cells at different stages were observed in testes of *Trappc2l*^−/−^ mice at 1 month after SSCs transplantation.

**
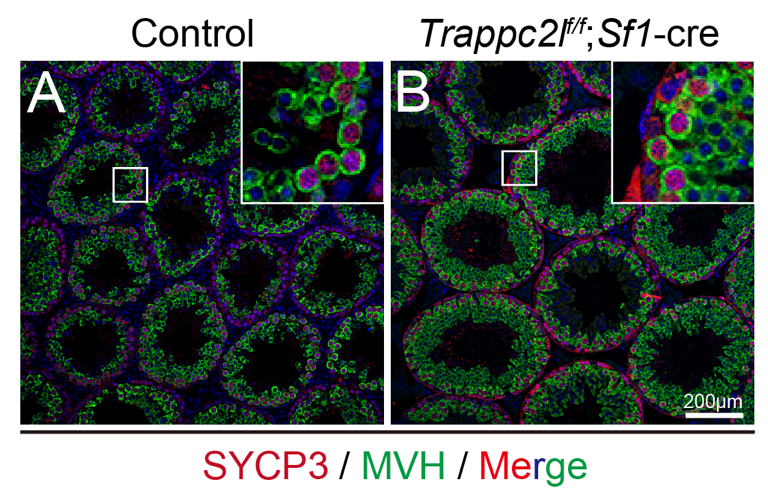
**

**Figure. S11.** **Inactivation of *Trappc2l* in Sertoli cells did not affect spermatogenesis.**

A-B. *Trappc2l* was knocked out in somatic cells by *Sf1*-cre. The expression of SYCP3 and MVH in control and *Trappc2l*^f/f^; *Sf1*-cre mice at 3W were examined by immunofluorescence. SYCP3 and MVH-positive germ cells were observed in testicular cords of both control and *Trappc2l*^f/f^; *Sf1*-cre mice.

**Supplementary table1.List of primers used in this study.**

**Supplementary table2.List of antibodies used in this study.**
